# Authors’ response

**DOI:** 10.1177/0272989X20946290

**Published:** 2020-08-26

**Authors:** Jolyn Hersch, Tessa Copp, Gemma Jacklyn, Jesse Jansen, Gerrit-Jan Liefers, Kirsten McCaffery, Anne Stiggelbout

**Affiliations:** Wiser Healthcare, Sydney School of Public Health, University of Sydney, New South Wales, Australia; Wiser Healthcare, Sydney School of Public Health, University of Sydney, New South Wales, Australia; Wiser Healthcare, Sydney School of Public Health, University of Sydney, New South Wales, Australia; Department of Primary Care, Maastricht University Medical Center, Netherlands; Department of Surgery, Leiden University Medical Center, Netherlands; Wiser Healthcare, Sydney School of Public Health, University of Sydney, New South Wales, Australia; Department of Biomedical Data Sciences, Leiden University Medical Center, Netherlands

Dear Editor:

We read with interest Gordon and Yaffe’s letter^1^ about our article, “Women’s Acceptance of Overdetection in Breast Cancer Screening: Can We Assess Harm-Benefit Tradeoffs?”^2^ We agree with them on some points but disagree on several others.

Quantifying overdetection is difficult, as is widely acknowledged. This is why—contrary to our correspondents’ claim^1^—we did not use one particular estimate of overdetection. Instead, we presented and explored responses to a wide range of overdetection levels reflecting prominent and disparate published estimates. Indeed, we explicitly informed participants that the “figures are hypothetical, we do not know what the exact numbers are in reality.”^2^ It seems somewhat inconsistent to us for our correspondents to first cite Helvie,^3^ who highlights the weaknesses of all efforts to quantify overdetection, and to then nevertheless seemingly endorse one estimate^4^ of the many published, which happens to lie at the low end of the spectrum. Moreover, restricting overdiagnosis estimates to invasive cancer would be inappropriate because screening also detects ductal carcinoma in situ (DCIS), which is also treated.

It is often assumed that breast screening results in women avoiding aggressive treatments because screen-detected cancers are typically found at an earlier stage and are therefore “easier” to treat. However, the randomized trials of mammography screening show the opposite.^5^ Overall, screening leads to more women having invasive treatments such as mastectomy and radiotherapy, not fewer. The Cochrane review on screening mammography found that women who were offered screening ended up with a 20% higher number of mastectomies than those not offered screening. The number of women undergoing radiotherapy was also higher in the screened groups.^5^ Even if chemotherapy and its substantial morbidities are avoided (and our article did acknowledge that screen-detected cancer is less likely to be treated with chemotherapy^2^), a diagnosis of cancer followed by lumpectomy, radiotherapy, and possibly hormone therapy can nonetheless cause physical and emotional harm to women and their families.^6–8^ The fear and other psychosocial consequences associated with being labeled with cancer, as well as the treatment impacts on quality of life, are very real.

Our correspondents remark upon our citation of work by Pappadis et al.^9^ These researchers interviewed women aged over 70 years, half of whom were over 75, concluding that “providing older women with descriptions of overdetection may not be sufficient to influence screening intentions.” We cited their study in our Discussion, as well as other previous studies, in order to put our findings into context with the existing literature. This by no means supports Gordon and Yaffe’s false claim that our “objective is to persuade women not to be screened.”^1^ Our aim was simply to investigate whether, and to what extent, women’s choices might be sensitive to variation in the magnitude of overdetection.

Our correspondents argue that “if the goal is to reduce overtreatment this is best accomplished not by avoiding screening with its potential of reducing mortality and morbidity, but instead to make careful decisions regarding the required aggressiveness of therapy.”^1^ We agree about the necessity of careful treatment decision making, incorporating a range of prognostic and patient factors. Ongoing research is testing the safety of active surveillance for some screen-detected lesions and will eventually provide evidence that may reduce overtreatment.^10^ At present, however, some treatment is virtually always recommended. Moreover, being newly diagnosed with cancer is a highly emotional experience, so careful and rational decisions about treatment are likely to be facilitated if women are made aware of the concept of overdetection before screening.

Gordon and Yaffe^1^ also assert that we “postulate that women who choose to be screened have made an irrational decision.” We did not postulate any such thing. An informed individual may rationally choose to screen or not to screen. We cited the “therapeutic illusion”^11^—a normal human phenomenon whereby people tend to attribute causality to actions taken, such as assuming that screening saved one’s life—as a potential contributor to the difficulty women face in making benefit-harm tradeoffs about screening.^2^ It is indeed impossible to ever know whether overtreatment has occurred, even in retrospect.^1^ We consider this all the more reason to bring the possibility to women’s awareness before they embark on a screening process that is designed to detect and facilitate treatment of very early cancers.

We reject our correspondents’ claim that our article reflects “a paternalistic position.”^1^ On the contrary: it is paternalistic to decide on women’s behalf that the benefit outweighs the harm, that women should screen, and that the facts about overdetection should be deliberately withheld from them while instead distributing persuasive and misleading material designed explicitly to maximize screening participation. Nowhere did we state that women shouldn’t screen; our argument is that women should feel supported to make a tradeoff of benefits and harms and then decide what is best for them. We want women to make an informed choice, rather than just uncritically accept screening. Of course, their choice could still be to screen, and this is perfectly appropriate if that is their informed decision.

We completely agree that “women should be able to decide what is best for them”^1^ by making their own subjective judgments based on the best available objective, evidence-based information. To this end, we have shown that explaining overdetection in a consumer-friendly decision aid increased women’s knowledge and enabled more women to make an informed choice about whether to screen or not.^12^

Our article contains no criticism of radiologists that could possibly warrant Gordon and Yaffe’s highly defensive response.^1^ We do, however, now take the opportunity to alert our correspondents to a potentially relevant systematic review of all studies that quantitatively assessed clinicians’ expectations of benefits and/or harms of any treatment, test, or screening test.^13^ The review found that clinicians rarely have accurate expectations of benefits or harms, with inaccuracies in both directions, but that clinicians more often underestimate rather than overestimate harms and overestimate rather than underestimate benefits.

Once again, we wholeheartedly endorse Gordon and Yaffe’s call^1^ for women to be supported in making and implementing the best choice for them—whether that is to screen or not to screen.
